# Lunacy Reform

**Published:** 1910-05-07

**Authors:** Forbes Winslow

**Affiliations:** 57 Devonshire Street, W.


*To the Editor of* The Hospital.

### LUNACY REFORM.

*To the Editor of* The Hospital.

Sir,—I am glad to see the insertion of my letter in your issue of to-day. Since I have agitated in the matter of Lunacy Reform I have received from the Borough Council of Birmingham a copy of the following resolution submitted to the Council and passed: "That in the opinion of this meeting there is urgent need of immediate legislation on the lines recommended in the report on the Feeble-minded, and that a copy of this resolution be sent to the Lord Chancellor, the Home Office, the Local Government Board, the Board of Education, the Lunacy Commissioners, and to the members of Parliament for the City of Birmingham." This was seconded by Bishop Gore, Bishop of Birmingham. I would also desire to state that inasmuch as it is more than five years since the Royal Commission was appointed, and that up to the present no reform in Lunacy matters has been made, or indeed is in contemplation, it is high time something was done. My resolutions, however, go beyond this resolution carried at Birmingham. This, however, is for reform as well. I am pleased to say since I wrote to you on April 13 I have obtained most influential support and I hope in a few days to announce particulars of the said meeting. Any medical man is welcome, and those who are opposed to legislation to benefit humanity can stay away.—Faithfully yours, Forbes Winslow.

57 Devonshire Street, W.,
April 30, 1910.

P.S.—With regard to the marginal note *re* the "Eminent Surgeon," I have not the slightest notion to whom you refer, and hence I am at liberty to comment on what you say. I maintain that any "gentleman who is now an officer in one of the medical services of the Crown" had no possible right to divulge the name of the "Eminent Surgeon" to you or anyone else. The names of certified lunatics are sacred. Again, possibly any blow inflicted by the "Eminent Surgeon" was only in self-defence, as he considered he had been shamefully wronged.—F. W.

